# Editorial: Innovating and expanding universal access to oral healthcare

**DOI:** 10.3389/froh.2022.1033469

**Published:** 2022-09-26

**Authors:** Bathsheba Turton, Callum Durward, Habib Benzian

**Affiliations:** ^1^ Henry M. Goldman School of Dental Medicine, Office of Global and Population Health, Boston University, Boston, MA, United States; ^2^ Faculty of Dentistry, University of Puthisastra, Phnom Penh, Cambodia; ^3^ WHO Collaborating Center, Quality Improvement and Evidence Based Dentistry, Epidemiology and Health Promotion, College of Dentistry, New York University, New York, NY, United States

**Keywords:** universal health care (UHC), basic package of oral care, inequalities (MeSH), global health, oral health

**Editorial on the Research Topic** Innovating and expanding universal access to oral healthcare

## Introduction

The state of oral health globally is alarming—almost half of the world's population suffer from oral diseases or conditions. Oral diseases are global public health challenges with serious impacts on health, health systems, the economy, and quality of life. Billions of people have no sustained and affordable access to oral health care (1). The high burden of oral diseases reflects widespread inequalities along socio-economic gradients; particularly affecting the most socially excluded and marginalized groups in society. Moreover, resources for prevention and treatment are inadequate in many countries; combined with insufficient protective population policies, and low availability of essential primary oral health care. Yet, private oral health care based on out-of-pocket payments or private insurance, is generally available for affluent population groups able to afford such services.

The Lancet Oral Health Series highlighted the global public health importance (and woeful neglect) of oral diseases, and “the need for a radically different policy agenda to tackle this major problem” (2, 3). Initiatives at the World Health Organization led to the adoption of a Global Strategy for Oral Health, and a renewed global commitment to end the neglect of oral health (4, 5). Central to this new momentum is the integration of essential oral health care in Universal Health Coverage (UHC), so that the most frequent oral problems can be addressed with a set of cost-effective interventions, including prevention and rehabilitation, in the context of primary health care and without financial hardship for patients.

This special issue presents articles on a range of topics related to expanding access to oral health care. In the past, efforts to strengthen oral health care systems were based on expanding models of traditional, technology-focused dental care. More realistic concepts focusing on essential oral health care, such as the WHO-supported Basic Package of Oral Care (BPOC), however, have seen only half-hearted implementation, lacked full health system support, rarely grew beyond a pilot stage, and were not well documents, resulting in limited impact. In addition, the persisting failure to integrate oral health in a primary health context in many health systems hampers universal access.

Innovative concepts and approaches are urgently needed to address these challenges. We believe that the selection of articles in this special issue align well with the six guiding principles of the WHO Global Strategy for Oral Health by offering real-life insights on how universal access to oral health care might be approached.

The paper from Burkina Faso by Clauss et al. found high levels of dental caries and periodontitis with very low levels of dental attendance and low use of fluoride toothpaste. Their paper reflects the situation in many LMICs where resources are in short-supply and oral health is not seen as a priority. The paper exemplifies the challenges of addressing the burden of oral disease, highlighting also the crucial importance of identifying people's needs as the basis for developing, testing, and implementing interventions and programs. The paper is an important demonstration about how data might be gathered to inform a public health approach to oral health (Global Strategy guiding principle 1: A public health approach to oral health).

The study by Susarla et al. on a large sample of mothers and children participating in a community-based oral health and nutrition program in five LMICs (Ecuador, El Salvador, India, Nepal, and Vietnam) between 2006 and 2015, found that oral healthcare was under-utilized by mothers and children as compared to general health services. The authors recommend integration of prevention- and treatment-oriented oral health care into primary health care, particularly prenatal care and child immunization services, as this could help increase access to oral healthcare and improve women's and children's oral health. This illustrates principle 2 of the WHO Global Strategy—Integration of oral health in primary health care.

A qualitative study from Nepal (Koirala et al.) investigates the effects of using a competency framework (CF) when implementing the BPOC. Health workers interviewed felt that the CF improved both their professional satisfaction and the quality of patient care. They recommended that CFs should be considered integral to the implementation of essential oral health care, along with opportunities for continuous professional learning. The study also highlights the need for enabling and supportive policies related to professional regulation and licensing when non-oral health personnel is taking roles in oral health care as part of a wider skill-mix and team approach. The work speaks to principle 3 around how a new oral health workforce might be supported (Principle 3: Innovative workforce models to respond to population needs for oral health).

The study by Turton et al. describes a unique school-based oral health program called Healthy Kids Cambodia. The program includes daily toothbrushing (DTB) and bi-annual application of Silver Diammine Fluoride (SDF). The authors conclude that the delivery of a package of care including both, DTB and SDF, has the potential to prevent adverse outcomes, such as dental infections in primary teeth affected by caries. The program is an example of tailoring effective setting-specific prevention and care for a particularly vulnerable population group (Principles 4 and 5: People-centered oral health care/tailored oral health across the life course). The paper also contributes to the body of evidence related to using SDF in a low-income country setting. SDF has recently been added to the WHO list of essential medicines for cost-effective management of dental caries among children and Adults.

The world is waking up to the potential for using such low cost strategies to control dental caries, as part of essential oral health care. SDF and other cost-effective interventions, such as fluoride toothpaste or sugar-sweetened beverage taxation, should be part of bundled approaches to essential oral health care and their integration in UHC. In line with the WHO Global Strategy on Oral Health's guiding principle 5 and the strategic objective 6 related to oral health research, we urge the oral health community to intensify applied research on all aspects related to expanding universal access to oral health care.
